# Russia Weaponization of Canada's Far Right and Far Left to Undermine Support for Ukraine

**DOI:** 10.1177/00207020241257635

**Published:** 2024-06-17

**Authors:** Brian McQuinn, Marcus Kolga, Cody Buntain, Laura Courchesne

**Affiliations:** Centre for Artificial Intelligence, Data and Conflict, Department of Politics and International Studies, 6846 University of Regina, Regina, Saskatchewan, Canada; DisinfoWatch and Macdonald-Laurier Institute, Ottawa, Ontario, Canada; College of Information Studies, 1068 University of Maryland, College Park, Maryland, USA; Center for International Security and Cooperation, Stanford University, Stanford, California, USA

**Keywords:** foreign influence operations, foreign influence operations, disinformation, social media, conflict, Russian disinformation campaign

## Abstract

This article details the Russian government's efforts to influence Canadians’ perceptions of the war in Ukraine. Specifically, we examined Russian information campaigns tailored to Canadian audiences on X (formerly known as Twitter) and the supportive ecosystems of accounts that amplify those campaigns. By 2023, this ecosystem included at least 200,000 X accounts that have shared content with millions of Canadians. We identified ninety accounts with an outsized influence. The vast majority of the influential Canadian accounts were far right or far left in orientation. These networks were among Canada's most prolific and influential political communities online. We determined this by comparing these networks’ potential influence to the online community engaging with Canada's 338 members of Parliament on X and a sample of twenty influential X accounts in Canada. The sophistication and proliferation of Canada-tailored narratives suggest a highly organized and well-funded effort to target Canadian support for Ukraine.

On 24 February 2022, Russia invaded Ukraine with overwhelming force, ending seventy years of peace in Europe.^1^ The war has devastated Ukraine, killed hundreds of thousands of Ukrainians and Russians, and displaced more than 15 million people.^2^ Russia's initial invasion failed, leading to the imposition of international sanctions and further isolating its president, Vladimir Putin. Despite being targeted, the sanctions’ impacts have been mixed, causing global spikes in food and oil prices.^3^ The result is a grinding war of attrition, with Russia now targeting civilian infrastructure,^4^ including hospitals and electricity grids—a strategy that violates international humanitarian law.^5^

Canada has deep ties to Ukraine and is home to one of the largest Ukrainian diaspora communities in the world. In response to the invasion, the Canadian government has provided over C$4.5 billion in humanitarian and military aid^6^ while strongly supporting NATO, the most potent military opposition to Putin.^7^ International assistance is essential to Ukraine's survival. Western governments have donated billions of dollars of technologically advanced weapons to Ukraine. The delivery of these weapon systems and non-lethal aid has significantly contributed to Ukraine's military capacity and the failure of Putin's military objectives.^8^

Public backing for Ukraine in Western countries is important for sustaining that aid. Putin recognizes this vulnerability and actively seeks to undermine support for Ukraine among Western democracies. One of his primary weapons in this fight is online influence campaigns pushed through Western-owned social media platforms such as X (formerly known as Twitter), YouTube, and Facebook.^9^ This paper details Russian efforts to influence Canadians’ perceptions of the war in Ukraine over the last two years, building on previous research within and outside Canada.^10^ Specifically, we study the influence campaigns tailored to Canadians, many built around Canadian themes or targeting specific Canadians.^11^ We contribute to these studies by identifying a previously unexplored Russian strategy in Canada: the weaponization of Canada's far-right and far-left movements to undermine international support for Ukraine.

## Methodology: Mapping Russian influence operations

The data we used to research online behaviours were acquired through X's academic research application programming interface (API) and a three-year qualitative study of Russian disinformation campaigns on social media. The quantitative analysis employs a network-based methodology developed in previous studies.^12^ This method relies on conflict experts to identify prominent accounts in a network, and then uses those accounts to map the broader ecosystem engaging with those accounts (see details below).^13^

The method used in this paper differs from previous Canadian studies, which relied on hashtags or key terms to identify social media posts related to the conflict.^14^ For example, Jean-Christophe Boucher and colleagues used key terms such as “NATO” to identify tweets associated with the invasion. They then studied only the accounts that identified themselves as being based in Canada. One weakness of this method is that many of the prominent accounts in the pro-Russian network in Canada do not identify, or no longer identify, as being based in Canada. Our researchers were familiar with many of the most influential accounts and noted that some removed their Canadian affiliation in the lead-up to the war or after it began. Consequently, our researchers were able to identify those accounts as being attributed to Canadian networks and include them in the study.

Another weakness of this other method is that Canadian-tailored content is diluted within all Russian English-language influence operations, most of which target Americans. This could lead analysts to conclude, for example, that Canada is not the target of much of this material. However, one would not expect Canada, given its relatively small population and geopolitical influence in the world, to be the primary target of Russian influence operations globally. For this reason, we mapped the social media network that engaged primarily with Canadian-tailored narratives to study the networks targeting Canada.

Other Canadian studies relied on data sets produced by international think tanks that were not tailored to Russian influence operations in Canada.^15^ By contrast, the method used here is a network-based methodology that recreates and then studies the network that is producing or amplifying Russian influence operations tailored to Canadians. We use this method as it allows us to track those networks on an ongoing basis and identify emerging narratives before they enter the broader social media ecosystem.

In preparation for this paper, the team conducted a three-year quantitative and qualitative study examining Russian disinformation campaigns across several Western democracies, including Canada, the US, and the EU.^16^ The qualitative researchers monitored overt Russian government actors such as Russian Television (RT), Sputnik News, and Kremlin spokespersons through open-source intelligence (OSINT) methods. These researchers then analyzed how specific stories were picked up, shared, and amplified in various pro-Russian ecosystems across different countries. To map the Canadian pro-Russian networks, the team selected forty-eight prominent X accounts promoting and amplifying Russian government-aligned narratives with Canada-related narratives. These accounts were then used to map these accounts’ supportive ecosystem: those accounts that either liked, reposted, or had material posted by the prominent X accounts. This network included more than 200,000 X accounts. Over 2 million tweets from these accounts were collected from 24 February 2021 to 31 January 2023. This period allowed the team to analyze the ecosystem for a year before the invasion to see how it changed before, during, and after the invasion. For instance, we were able to show how the network and its narratives exponentially increased their messaging in the weeks before military actions began, showing its proactive role in the invasion (see findings below for more details).

To measure the potential influence of the pro-Russian ecosystem, we identified the accounts most central to the network and compared them to other prominent networks in Canada. We identified the central accounts by using a centrality metric across all accounts. This centrality metric, PageRank, is used to identify influential web pages.^17^ It has been used to study social networks, especially around conspiracy theories.^18^ Using this centrality metric, we found ninety accounts that were disproportionally central to the network.

In order to test how influential or significant these accounts were in the broader Canadian social media landscape, we compared them to two other sets of accounts: a) the online community that engages with Canada's 338 members of Parliament (MPs) on X; and b) a list of Canada's most influential X users. These two sets were compiled through published lists.^19^ These baselines helped assess how influential and prolific the pro-Russian accounts were by comparing their account follower count, tweet frequency, and accounts that they followed. Specifically, we compared the number of tweets produced by accounts (“Tweet Count”), how many followers each account had (“Follower Count”), how many accounts each followed (“Following count”), and how many lists contained a given account (“Listed Count”). The latter factor is a proxy for visibility or expertise, as being in more lists indicates the account is more well known.

## Analysis: X's Russian-aligned ecosystem in Canada

We studied Canada's supportive pro-Kremlin ecosystem of more than 200,000 accounts, as recent research has shown that foreign influence operations are collaborative undertakings, requiring a supportive online network to spread and amplify messages.^20^ The analysis allowed us to examine how Canadians without strong political views were central to this supportive network, which is important information for designing effective interventions. The analysis provided compelling evidence for the following conclusions:

### Russian influence operations are weaponizing Canada's far right and the far left

The analysis mapped the supportive ecosystem that amplifies narratives pushed by the Russian government and its proxies on X. As we described earlier in the methodology section, this ecosystem included at least 200,000 accounts that retweeted or liked these narratives. Network mapping software then identified the ninety most influential accounts in the ecosystem. Our analysts were familiar with most accounts, having tracked Russian influence operations in Canada for the last three years. The team removed thirty-three accounts for which the user's location could not be determined. Of the remaining accounts, the largest group of users were based in Canada (47 percent), with the US (23 percent), Russia (19 percent), UK (7 percent), Austria (2 percent), and Australia (2 percent) making up the remainder. The network comprises accounts advancing Canadian-related content. Nevertheless, it would be expected that those messages would be picked up and amplified by far-right and far-left networks in other countries, which is why they would figure prominently in the results.

The team then reviewed the accounts based in Canada and found that the overwhelming majority (92.6 percent) belonged to either far-right or far-left voices. Categorizing accounts as either “far right” or “far left” is a subjective assessment. However, most of the accounts in this study were well-known to our team, making the determination relatively straightforward. For the remaining accounts, our analysts studied an account's history for evidence of support for specific narratives and slogans promoted or amplified by groups on the far left and far right.^21^

**Figure 1.**
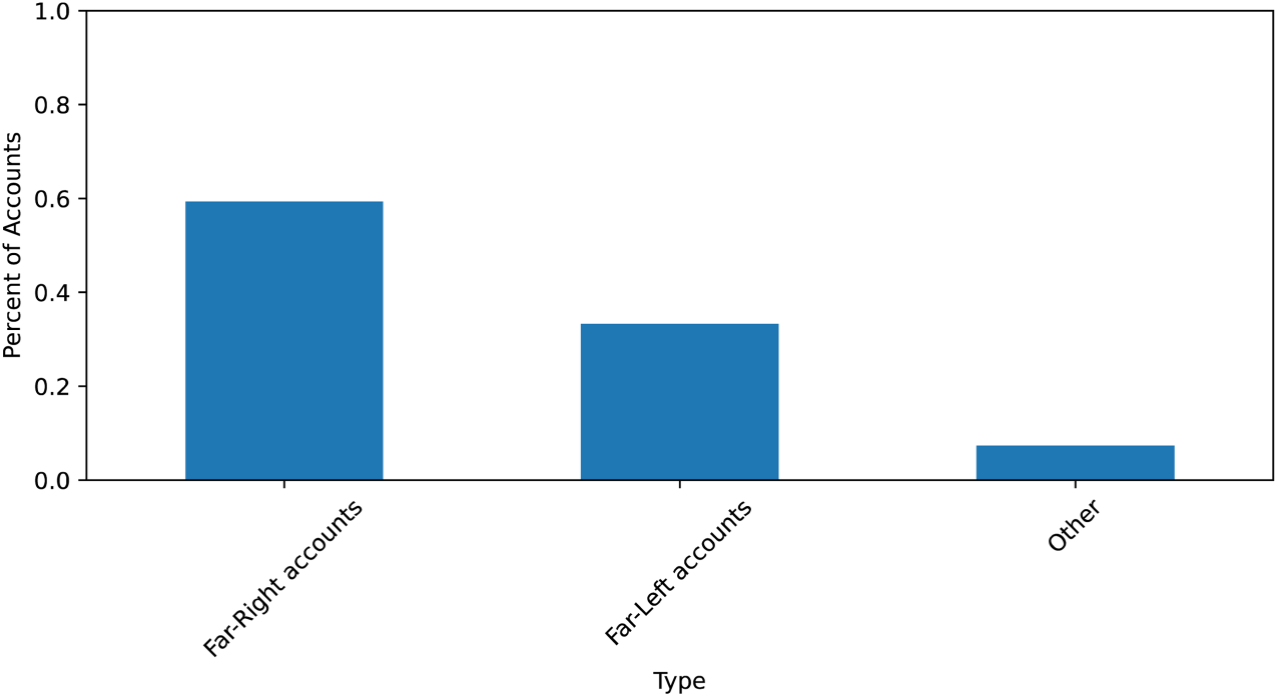
The political orientation of the core Russian-aligned accounts.

In total, nine accounts were characterized as far left (33.3 percent), while sixteen accounts were categorized as far right (59.3 percent). See Figure 1. The remaining accounts (7.4 percent) could not be classified along the traditional right and left political spectrum as they often expressed views from both extremes of the political spectrum.^22^ These accounts may signal the emergence of new political orientations that do not easily fit along the traditional right-left political spectrum.

The centrality of these accounts to the Russian influence operations in Canada provides compelling evidence that far-right and far-left communities have been co-opted to undermine Canadian support for Ukraine. These results mirror the findings of a 2016 study in the US that mapped the online communities for and against Black Lives Matter protests in 2016.^23^ In that case, Russian-aligned accounts amplified polarization by playing a central role in both the far-right and far-left communities online.

### Far-right and far-left networks are among the most active online political communities

One of our primary goals in this report was to measure the relative size and reach of the networks amplifying Russian influence narratives in Canada. To assess the networks’ potential influence, we first identified the supportive ecosystem engaging with these information campaigns.

The Canadian pro-Russian ecosystem had at least 200,000 accounts on X, made up of two distinct networks. Only when we reviewed accounts central to these two ecosystems did we realize that the two networks corresponded to the far-right or far-left communities in Canada. As described above, we identified the central accounts by calculating the PageRank centrality metric across all accounts.^24^ Using this centrality metric, we found that its value drops off quickly after the core ninety accounts. As we reviewed the ecosystem's core accounts, our analysts determined that the users in Canada were among Canada's most prominent far-right and far-left voices.

To contextualize the potential influence of the pro-Russian ecosystem, the team identified two comparable sets of accounts. The first network mapped the X ecosystem for Canada's 338 MPs who have X accounts. This network served as a comparison of a politically oriented online community in Canada. For the second network, the team identified the most influential X accounts in Canada to serve as a benchmark against the pro-Russian networks we had identified. The data set was created by collecting X data geo-located to Canada, as identified by X's geo-location metadata and applying the same PageRank-based approach to identify the most influential accounts. These included accounts such as the Canadian Broadcasting Corporation (CBC) and Prime Minister Justin Trudeau. The three sets of accounts were then compared by examining, among other comparisons, the number of tweets they produced, the number of times they were retweeted, the number of accounts that followed each, and the number of accounts they followed.

**Figure 2.**
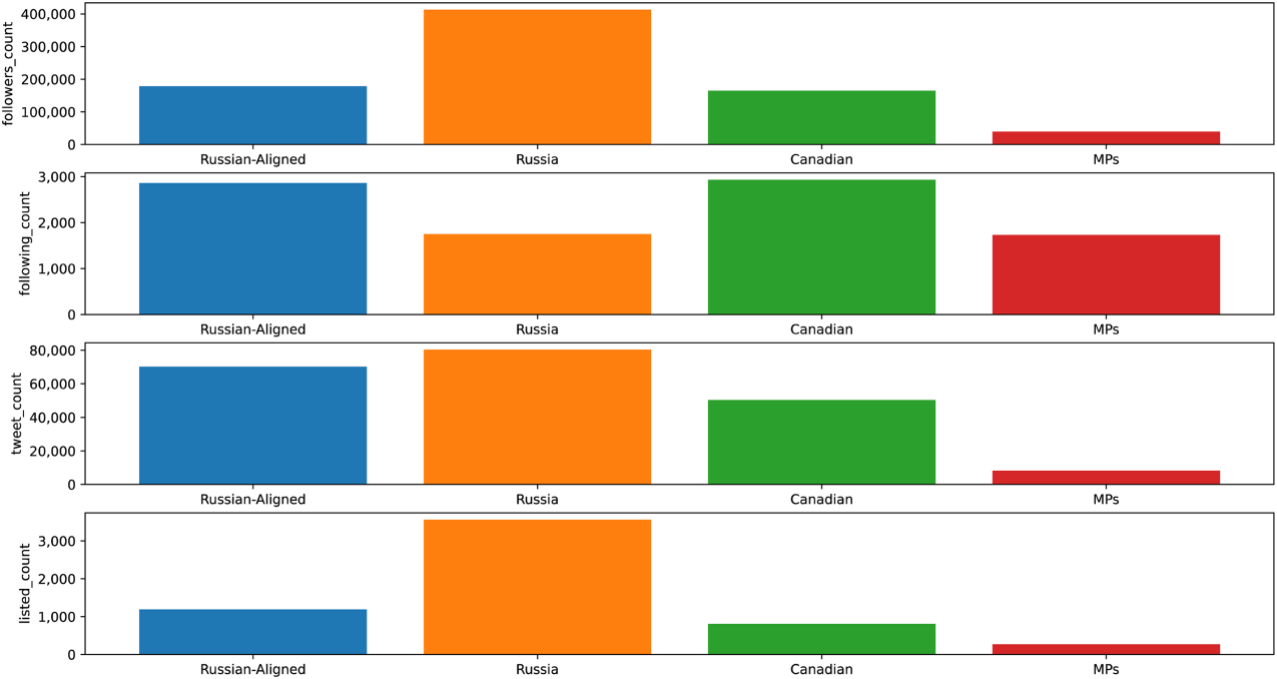
Comparing Canadian Russian-aligned accounts, Canadian MPs, and the most prominent X accounts in Canada.

In comparison to the 338 MPs, the Canadian Russian-aligned accounts had comparable followers, followed three times more accounts, produced twenty-seven times more tweets, and had three times more engagement (see Figure 2). However, this analysis does not account for the ultimate influence of those ideas on Canadians’ views of Ukraine. Still, as a network, it is among Canada's most active political communities. Measuring the Russian-aligned accounts compared to the most influential accounts in Canada of any kind, they produced four and a half times more content (450 percent), followed almost twice as many accounts, but had only a quarter (27 percent) of the followers. The analysis showed that the Russian-aligned posts were engaged with more often when accounting for the size of their audience.

### Average Canadians enabled Russian influence operations

The most active accounts—those driving much of the traffic—were a tiny percentage of the ecosystem (far less than 1 percent). Crucially, this meant that the vast majority of X accounts in the supportive ecosystem (83.4 percent) amplifying pro-Russian narratives were average Canadians, defined as any user with fewer followers than the average member in the pro-Russian ecosystem.^25^

**Figure 3.**
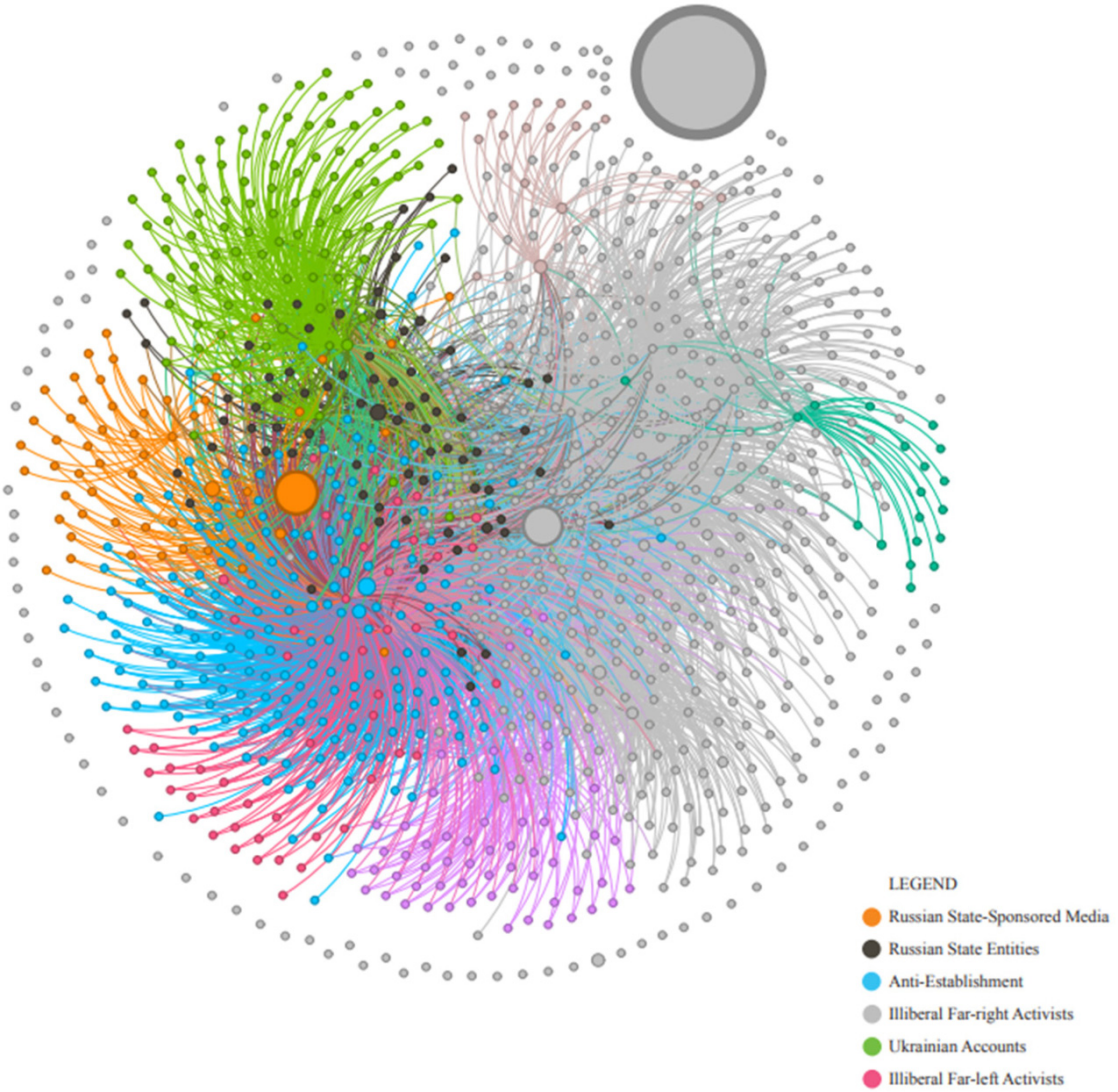
The Russian-aligned ecosystem.

One insight from this study, which is supported by other research, is how a small network of active pro-Putin accounts can co-opt and engage passive followers.^26^ Without this tacit and usually unknowing support by average Canadians, such pro-Putin narratives would not be amplified in Canadian online spaces to the extent that they are (see Figure 3).^27^

### Russian disinformation campaigns use content tailored to Canada

Our findings confirmed previous research that Russian influence operations use Canadian-tailored content.^28^ The most obvious examples include influence campaigns targeting specific Canadians. For example, one of the most consistent targets was Canadian deputy prime minister Chrystia Freeland, a strong supporter of Ukraine.^29^ Other narratives blamed Canadian policies toward Ukraine for various global ills, including inflation and global food shortages, or promoted Russian motivations for initiating the war. Studying content with Canadian-related material helped distinguish the supportive ecosystem in Canada from accounts in the US. However, it was clear from the network mapping and qualitative assessment that significant sharing occurs between the US and Canadian ecosystems.

Our research confirmed other studies’ findings that new influence narratives targeting Canada emerge every week.^30^ These influence campaigns often produce variations of existing targets or themes but they also respond quickly to political and military events in Canada, Europe, Russia, and Ukraine. The sophistication and proliferation of these Canada-tailored narratives suggest a highly organized and well-funded effort to target Canadian support for Ukraine.

The narratives pushed by accounts linked to Russian interests include the following narratives, many of which have been identified by previous research:

- “Canada's foreign policy is controlled by Ukrainian Canadians.”
- “Canadian sanctions are responsible for inflation and rising energy costs.”
- “Canadian sanctions are responsible for growing global food shortages.”
- “If Canadians want to cooperate with Russia on climate and Arctic issues, then we must return to diplomacy.”
- “Ukraine is corrupt and doesn’t deserve our support.”
- “Russia is de-Nazifying Ukraine.”
- “NATO is responsible for the war.”
- “Western support for Ukraine should stop because it will cause nuclear Armageddon.”

### Russian information operations spiked in the three months before the invasion

**Figure 4.**
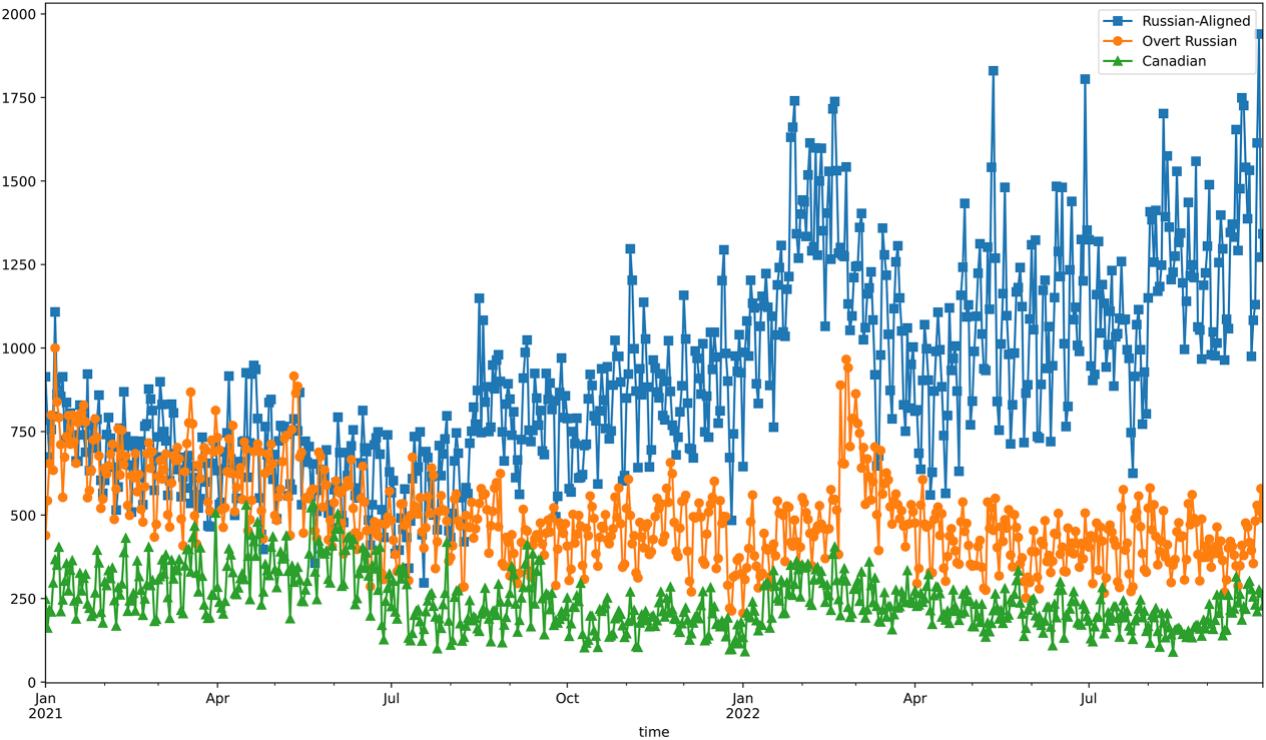
Comparing daily Russian tweet production.

The study examined tweets from Russian-aligned accounts for a full year before the 24 February 2022 invasion. This analysis allowed the researchers to establish baseline assessments of the ecosystem that would later be central to amplifying Russian government narratives. Figure 4 tracks the daily tweet production within the three X ecosystems described before: a) the online political community engaging with Canada's 338 MPs; b) twenty of Canada's most influential X accounts; and c) a Russian aligned-network focused on Canadian content.

Our analysis showed that the production of tweets in the Russian-aligned accounts quadrupled in the three months before the invasion, providing compelling evidence of a premeditated campaign to shape Canadians’ views before the invasion. The results also highlight a steady increase of approximately 8 percent per month in the tweet production of that network since the invasion on 24 February 2022.

## Recommendations to combat Russian disinformation in Canada

We have highlighted the prevalence and reach of the Russian government's efforts to undermine Canadian support for Ukraine. These efforts began months before the invasion and are increasing as the war continues. There are many government and civil society organizations combating Russian influence operations. Based on our experience with many of these programmes and efforts, we recommend the following to better combat Russian influence operations in Canada:

### Strengthen government systems of oversight and information sharing

- *Increase efforts to ensure that elected officials at the federal and provincial levels are aware of the extent and strategies of Russian influence operations in Canada*. This would include regular briefings to elected representatives and government officials to help ensure that they do not inadvertently amplify or normalize pro-Russian narratives.
- *Establish an all-party parliamentary committee for defending democracy*, which would include members from each political party in Parliament, members of the Security and Intelligence Threats to Election (SITE) Task Force, and representatives from civil-society experts and the media.
- *Form a national council for democracy* that would monitor emerging threats and foreign influence narratives. The group would alert social media and government representatives to these threats and possible responses. It would also formulate a national whole-of-society strategy to foster long-term national resilience against malicious foreign information and influence operations.
- *Ensure greater intergovernmental coordination and collaboration*. Several Canadian federal and provincial ministries, departments, and agencies have ongoing responsibilities and programmes that monitor, analyze, or expose foreign information operations. Establishing an overarching committee within the government could improve intergovernmental coordination and input from civil society actors and the media. This committee could be responsible for engagement within the national council for democracy (see recommendation 1c) and provide data to the all-party parliamentary committee for defending democracy (see recommendation 1b).

### Increase international coordination and collaboration

The Canadian government should coordinate efforts to monitor and expose information operations targeting its allies and collectively advocate for more robust self-regulation by social media companies. Many of Canada's allies have significant experience in defending against the threat of malicious foreign information operations, including the Baltic States, Finland, Sweden, and Taiwan.

### Increase support to schools, civil society, and research organizations

Support efforts by civil society and researchers to monitor, analyze, and expose foreign disinformation narratives. Greater awareness of these narratives, of the reasons foreign actors use tactical narratives, of who they target, and of the consequences of such narratives will lead to greater long-term resilience against them. Social and digital media literacy are essential skills for Canadian society. It is especially crucial that children develop the necessary skills to inoculate them against information operations.

### Social media companies should provide researchers with greater access to data

Researchers’ capacity to study foreign information campaigns in Canada is dramatically limited by the restrictions placed on academics studying most social media platforms. Governments should incentivize these platforms to provide access to researchers and civil society organizations. The quantitative research in this report is based on X data because of its publicly available API. While we found evidence of Russian influence operations on other social media platforms (including Facebook, Instagram, and YouTube), we were limited in our ability to examine its scope. Social media companies that are serious about understanding the misuse and weaponization of their platforms by violent and extremist actors must equip researchers with the tools required to quantify the breadth and extent of the problem.

### Stay ahead of pro-Russian networks through real-time monitoring and learning

Current moderation efforts tend to be based on community standards and policies, often aiming to manage violations rather than preventing or minimizing them. Given that malicious networks typically find ways to circumvent rules and restrictions, social media companies can retain their edge by focusing on building—and sustaining—a continuous learning system that can monitor and adapt more quickly than nefarious actors can. This shift in moderation techniques involves reducing the reliance on reactive strategies that respond to single inappropriate activities and instead increasing the capacity to track foreign interventions through actor-centred approaches.

## Conclusion

All signs suggest that the war in Ukraine will continue for the foreseeable future. Ukraine's ability to fight will heavily depend on continued Western support. It is almost certain that efforts by the Russian government and its proxies to undermine support for Ukraine will only increase in the coming months. It is also likely that social media platforms will continue to be the main avenue for influence operations, disrupting authentic political debate in Canada.

Canadians should be able to discuss and debate the nature of support to Ukraine without malicious influence by foreign states. It is therefore crucial that the Canadian government, civil society, and social media platforms work together to reduce foreign influence operations. This should include additional resources for monitoring foreign interference. For instance, creating Russia and China teams within the Rapid Reaction Mechanism at Global Affairs is a welcome step toward achieving this. However, a whole-of-society approach is required, as is greater coordination and cooperation between relevant departments.

A crucial element of that approach involves better tools and methods to map the evolving ecosystems in Canada that are co-opted—willingly or not—by foreign influence operations to ensure that their impact can be minimized. These tools include human-in-the-loop AI solutions that draw upon human analysis of the rapidly changing threat combined with the reach and pattern recognition of specialized AI algorithms. Together, they can contribute to timely and effective monitoring, exposing, and countering foreign narratives that are injected or amplified in our information space, and help build long-term public resilience against these narratives.

## Notes

1. The dissolution of Yugoslavia (1992–1995) was a devastating chapter in European history. However, it was a civil war and while it was internationalized, it did not involve an inter-state conflict. See Steven L. Burg and Paul S. Shoup, *The War in Bosnia-Herzegovina: Ethnic Conflict and International Intervention* (Armonk: M.E. Sharpe, 1999).

2. BBC, “Ukraine war: US estimates 200,000 military casualties on all,” 10 November 2022, https://www.bbc.com/news/world-europe-63580372 (accessed 6 May 2024); Erol Yayboke, Anastasia Strouboulis, and Abigail Edwards, “Update on forced displacement around Ukraine,” Critical Questions, Center for Strategic and International Studies, 3 October 2022; “Ukraine: Russian attacks on critical energy infrastructure amount to war crimes,” Latest News, Amnesty International, 20 October 2022, https://www.amnesty.org/en/latest/news/2022/10/ukraine-russian-attacks-on-critical-energy-infrastructure-amount-to-war-crimes/ (accessed 6 May 2024).

3. Peterson K. Ozili, “Global economic consequence of Russian invasion of Ukraine,” 2022, https://dx.doi.org/10.2139/ssrn.4064770; Susan Hannah Allen, “The uncertain impact of sanctions on Russia,” *Nature Human Behavior* 6, no. 6 (2022): 761–762.

4. James Kariuki, “Russia's systematic attacks on Ukrainian civilian infrastructure are unacceptable, and must end,” Statement by Ambassador James Kariuki at the Security Council briefing on Ukraine, Government of the United Kingdom and Northern Ireland, 23 November 2022.

5. Lara Hakki, Eric Stover, and Rohina J. Haar, “Breaking the silence: Advocacy and accountability for attacks on hospitals in armed conflict,” *International Review of the Red Cross* 102, no. 915 (2020): 1201–1226.

6. “Canada disburses $450 million in loans to Ukraine,” News Release, Department of Finance Canada, 17 August 2022; “Prime minister announces additional military assistance for Ukraine and additional sanctions against Russia,” News Release, 14 November 2022, Office of the Prime Minister, Government of Canada.

7. The North Atlantic Treaty Organization (NATO) is a defence pact established after World War II between the United States, Canada, and Europe. For further details see https://www.nato.int.

8. Andrew E. Kramer, “With Western weapons, Ukraine is turning the tables in an artillery war,” *The New York Times*, 1 November 2022.

9. W. Lance Bennett and Steven Livingston, “The disinformation order: Disruptive communication and the decline of democratic institutions,” *European Journal of Communication* 33, no. 2 (2018): 122–139.

10. Ahmed Al-Rawi, “How did Russian and Iranian trolls’ disinformation toward Canadian issues diverge and converge?” *Digital War* 2 (2021): 21–34; Barry Cartwright, Richard Frank, George Weir, and Karmvir Padda, “Detecting and responding to hostile disinformation activities on social media using machine learning and deep neural networks,” *Neural Computing and Applications* 34, no. 18 (2022): 15141–15163; Aengus Bridgman, Mathieu Lavigne, Melissa Baker, Thomas Bergeron, Danielle Bohonos, Anthony Burton, Katharine McCoy, et al., “Mis- and disinformation in the 2021 Canadian federal election,” Canadian Election Misinformation Project, OSF Preprints, 2022; Jean-Christophe Boucher et al., “Disinformation and Russian-Ukrainian war on Canadian social media,” SPP Briefing Paper, University of Calgary, June 2022, 15–16; Philip Mai, Alyssa N. Saiphoo, Anatoliy Gruzd, and Felipe Bonow Soares, “Russian propaganda is making inroads with right-wing Canadians,” *The Conversation,* 17 July 2022; “Disinformation roulette: The Kremlin's year of lies to justify an unjustifiable war,” Global Engagement Center report, United States Department of State, 23 February 2023; Ahmed Al-Rawi & Abdelrahman Fakida, “The methodological challenges of studying ‘fake news,’ *Journalism Practice* 17, no. 6 (2023): 1178–1197.

11. For an example, see Ashley Burke and Sylvia Thomson, “’I'm alive': Former Canadian Forces sniper debunks rumours of his death in Ukraine,” CBC News, 22 March 2022, https://www.cbc.ca/news/politics/wali-alive-despite-russian-disinformation-1.6393191# (accessed 7 May 2024).

12. Laura Courchesne, Bahar Rasikh, Brian McQuinn, and Cody Buntain, “Powered by Twitter? The Taliban's takeover of Afghanistan,” Centre for Artificial Intelligence, Data, and Conflict, University of Regina, 2022.

13. Samuel Stehle, “Mapping mediascapes: Exploring the semantic and geographic spaces of Catalonia's 2015 independence movement through digital news,” *The Professional Geographer* 73, no. 1 (2021): 150–159.

14. Darren L. Linvill and Patrick L. Warren, “Troll factories: The internet research agency and state-sponsored agenda-building,” Resource Centre on Media Freedom in Europe, OBC Transeuropa, Trento, June 2018.

15. Ahmed Al-Rawi, “How did Russian and Iranian trolls’ disinformation toward Canadian issues diverge and converge?” *Digital War* 2 (2021): 21–34.

16. For further information, see the “About” section of Disinfo Watch: https://disinfowatch.org/about/.

17. Yu Zhang, Zhaoqing Wang, and Chaolun Xia, “Identifying key users for targeted marketing by mining online social network,” 2010 IEEE 24th International Conference on Advanced Information Networking and Applications Workshops, Perth, Australia, 2010, 644–649, https://doi:10.1109/waina.2010.137.

18. Daniela Mahl, Jing Zeng, and Mike S. Schäfer, “From ‘Nasa lies’ to ‘reptilian eyes’: Mapping communication about ten conspiracy theories, their communities, and main propagators on Twitter,” *Social Media and Society* 7, no. 2 (2021): 1–12, https://doi.org/10.1177/20563051211017482.

19. For this work, we have used the published Twitter (X) list provided by the Canadian Jewish Political Affairs Committee, https://twitter.com/i/lists/224955374.

20. Kate Starbird, Ahmer Arif, and Tom Wilson, "Disinformation as collaborative work: Surfacing the participatory nature of strategic information operations," *Proceedings of the ACM on Human-Computer Interaction* 3, no. 127 (2019): 1–26.

21. We drew upon various approaches, including: Valentine Crosset, Samuel Tanner, and Aurélie Campana, “Researching far right groups on Twitter: Methodological challenges 2.0,” *New Media & Society* 21, no. 4 (2019): 939–961; Andrew Crosby, "Policing right-wing extremism in Canada: Threat frames, ideological motivation, and societal implications," *Surveillance & Society* 19, no. 3 (2021): 359–363; Thomas J. Holt, Mattisen Stonhouse, Joshua Freilich, and Steven M. Chermak, "Examining ideologically motivated cyberattacks performed by far-left groups," *Terrorism and Political Violence* 33, no. 3 (2021): 527–548. For further analysis on categorizing extremism, see John M. Berger, *Extremism* (Cambridge: MIT Press, 2018).

22. For a similar discussion related to political extremism in Europe, see Cas Mudde, *On Extremism and Democracy in Europe* (Abingdon and New York: Routledge, 2016).

23. Starbird, Arif, and Wilson, “Disinformation as collaborative work.”

24. Zhang, Wang, and Xia, “Identifying key users for targeted marketing by mining online social network.”

25. On average, members of the pro-Russian ecosystem had an average number of 1,318 followers. The remaining 17 percent with more than the average had approximately 6,477 followers on average, with a maximum of 800,175.

26. Starbird, Arif, and Wilson, “Disinformation as collaborative work.”

27. Andrew Weisburd, Clint Watts, and J.M. Berger, "Trolling for Trump: How Russia is trying to destroy our democracy," War on the Rocks, 6 November 2016, https://warontherocks.com/2016/11/trolling-for-trump-how-russia-is-trying-to-destroy-our-democracy/ (accessed 7 May 2024).

28. Boucher et al., “Disinformation and Russian-Ukrainian war on Canadian social media.”

29. The focus on Ms. Freeland is likely a result of her prominent role in the government, previous public support for Ukraine, and family ties to Ukraine. But it is also likely that Ms. Freeland is a target because of her potential candidacy as NATO's next secretary-general. See Murray Brewster and Alexander Panetta, “Chrystia Freeland has a ‘legitimate shot’ at top NATO Job, expert says,” CBC, 9 September 2022; Steven Erlanger, “Who will be NATO's next chief? The race is on,” *The New York Times*, 4 November 2022.

30. For example, see Madeline Roache, Sophia Tewa, Alex Cadier, et al., “Russia-Ukraine Disinformation Tracking Center: 358 websites spreading war disinformation and the top myths they publish,” NewsGuard, 21 February 2023, https://www.newsguardtech.com/special-reports/russian-disinformation-tracking-center (accessed 7 May 2024). See also the EUvsDisinfo site at https://euvsdisinfo.eu/.

